# Prevalence and perception of cigarette smoking among out of school adolescents in Birnin Kebbi, North-western Nigeria

**DOI:** 10.11604/pamj.2018.30.304.16641

**Published:** 2018-08-31

**Authors:** Godwin Jiya Gana, Suleiman Hadejia Idris, Kabiru Sabitu, Mansur Oche Oche, Aisha Ahmed Abubakar, Patrick Mboya Nguku

**Affiliations:** 1 Nigerian Field Epidemiology and Laboratory Training Program, 50, Haile Selassie Street, Asokoro, Abuja, Nigeria; 2 Department of Community Health, Usmanu Danfodiyo University Teaching Hospital, Sokoto, Nigeria; 3 Department of Community Medicine, Faculty of Medicine, Ahmadu Bello University, Zaria, Nigeria

**Keywords:** Adolescents, peer smoking, tobacco, cigarettes, youth, addiction, NCDs

## Abstract

**Introduction:**

cigarette smoking is on the increase among adolescents' especially in developing countries and is a leading cause of premature morbidity and mortality worldwide. Adolescents incorrectly perceive that cigarette smoking is less risky than other behaviors such as alcohol consumption and drug use. This study assessed the prevalence and perception of out of school adolescents on cigarette smoking in Birnin kebbi metropolis of Kebbi state.

**Methods:**

This is a cross-sectional mixed methods study. For quantitative data, respondents were selected using two-staged sampling technique. Semi structured interviewer-administered questionnaire was used. Univariate and bivariate analysis was done as appropriate using Epi-info software. Focus Group Discussion guide was used for qualitative data collection. Narrative synthesis was used for qualitative data analysis.

**Results:**

The proportion of respondents who had ever smoked cigarettes was 32.2% while 20.8% currently smoke. Most of the respondents (84.1%) perceived that cigarette smoking was harmful and that passive smoking was dangerous to their health (70.8%). Smokers had a significantly lower risk perception about smoking compared to non-smokers (p < 0.005).

**Conclusion:**

The prevalence of cigarette smoking among out of school adolescents is high with smokers having lower risk perception compared to non-smokers. There is an urgent need to create awareness about the specific dangers associated with cigarette smoking, the transient nature of its perceived benefits and the fact that the risks associated with smoking are severe.

## Introduction

Smoking is one of the most common forms of recreational drug use. In recent times, tobacco smoking appears to be the most popular form of smoking ahead of pipes, cigars and chewing tobacco and is practiced by over one billion people worldwide [1]. During the 20^th^ century, cigarettes became the predominant form of tobacco used across the world and ushered in the global lung cancer epidemic [2, 3]. Different patterns of diffusion of cigarette smoking across world cultures have been described, noting the early adoption by Western high-income countries and the slower adoption in many lower-income and middle-income countries [2]. According to the "Global youth tobacco survey" approximately 15% of adolescents smoked cigarettes in 4 of 29 sites in African region, 13 of 38 sites in American region, one of 23 sites in Eastern Mediterranean region, 15 of 29 sites in European region, one of 10 sites in South East Asian region, and 13 of 22 sites in West Pacific region. A marginal yearly increase of 3% in cigarette smoking prevalence has been reported for developing countries [4]. Young persons are being introduced to cigarette smoking at an earlier age and because of its addictive nature become hooked on it. Variable rates of increasing smoking prevalence have been reported in Nigeria which was found to be dependent on the settings-either rural or urban [5-8]. Some studies in Nigeria have reported tobacco smoking prevalence of between 3.4% and 17.1% [9-12]. The prevalence among secondary school students is approximately 9% [13] with a mean lifetime smoking prevalence of 7.2% to 42.9% [14]. The increasing rate of cigarette smoking is been consolidated by the various apparently successful marketing strategies of tobacco companies and the poor awareness about the dangers associated with smoking [15]. Cigarette smoking has been associated with an extensive list of health disorders as well as reduction of life expectancy [16]. On the average cigarette smokers lose about 15 years of their life [16] and an estimated 4 million cigarette smokers die worldwide annually [17]. Many researchers have also reported that cigarette may be the first drug to be used by adolescents in a sequel that may include alcohol, marijuana and hard drugs, individuals who are not smoking cigarette by the age of 20 are unlikely to become smokers [16, 18-20].

Multiple levels of influence i.e individual, interpersonal, community have been found to affect behaviour change especially amongst adolescents [21]. One common factor that operates at all these levels of influence is the psycho-social construct of the behavior in question, whether in the forms of personal beliefs, opinions, subjective norms, or social norms. This suggests that perceptions about smokers (at the personal, interpersonal and community levels) constitute one of the factors that may influence adolescents to adopt or reject smoking behavior [21]. Research has shown that perception of the risks and benefits of smoking are associated with cigarette smoking in adolescents [22]. Specifically, adolescents who smoke report lower perceptions of risk, including lower estimates of lung cancer, mortality risk, and getting into trouble, [22-25] as well as higher perceptions of benefits of smoking than adolescents who have never smoked [23, 26-29]. Perceptions of risks and benefits have been shown to increase the chance of adolescent smoking initiation by between two to four times [23]. A systematic review revealed that youths beliefs about the likelihood of addiction, health risks, and consequences of smoking are associated with future smoking behavior [30]. Adolescents' perceptions are expected to change over time as a result of making new friends and social interactions that influences their decision-making abilities. They tend to have poor decision making and risk-judging skills, leading them to believe they are invulnerable to harm [23]. This is made worse by adolescents who smoke and tend to influence their non-smoker friends to adopt the practice. There is however a dearth of research among out of school adolescents as most researches including the "Global Youth Tobacco Survey" [17] were conducted among secondary school adolescents. Out of school adolescents are usually understudied and underrepresented [31] and therefore evidence from researches about them are not usually considered when policy makers make decisions that concerns them. This study was conducted among out of school adolescents in Birnin kebbi metropolis of Northwestern Nigeria where these adolescents form a significant proportion of the total adolescent population with the intent that its findings would add to the body of knowledge about cigarette smoking among out of school adolescents and it will inform better decision making in matters of cigarette smoking that relates to them.

## Methods

**Study area:** Birnin Kebbi is located in Kebbi state, North-western Nigeria. The main preoccupation of the inhabitants is farming and fishing. Adolescents are found in their numbers at market areas, motor parks, building construction sites and on busy streets hawking goods and roaming around waiting to be beckoned on to do one menial job or the other. Cigarettes are sold at corner shops on busy streets, motor parks, shops/stalls at entrance to marketplaces, shops serving the state secretariat office complex and other busy areas. There is no health facility offering adolescent health services nor a functional youth friendly centre in Birnin kebbi.

**Study population:** Adolescents aged 10-19 years who have never been to school or have been out of school for at least 6 months preceding the survey and reside in Birnin Kebbi metropolis.

**Study design:** A cross-sectional mixed methods descriptive study design.

**Sample size estimation:** The minimum sample size was calculated using power of 80% and level of precision set at 0.05. Allowing for non-response, the eventual sample size was 342.

### Sampling technique

*Quantitative component*: Multistage sampling method was conducted. Stage 1: simple random sampling was used to select five (5) of the ten (10) wards which had adolescent aggregation points by balloting in the metropolis. Stage 2: systematic sampling technique was used for selecting the participants. Proportional allocation of participants per aggregation area in the selected wards was done as follows. Number of participants from site A = Average number of adolescents in site a total number of adolescents in all sites x Sample size At this stage, systematic sampling was used to recruit participants from each aggregation site. To do this a sampling frame of each aggregation site was gotten. This was used to calculate the required sampling interval as follows: Sampling interval = Sample frameProportional sample size for the aggregation site This gave the selection interval of participants to be recruited into the study from each study site. However to get the first participant simple random sampling was used to choose between 1 and the calculated sampling interval.

*Qualitative component*: convenience sampling was used to recruit participants for Focus Group Discussions (FGDs).

### Instruments of data collection

*Quantitative data*: a semi-structured, interviewer-administered questionnaire adapted from validated instruments used in previous surveys [32, 33] was used for data collection.

*Qualitative data*: a FGD guide was used for data collection so that more information on the various aspects of smoking perception among out of school adolescents could be explored.

**Methods of data collection:** Data was collected over five week period.

*Quantitative data*: the questionnaire was pre-tested in a neutral area (Sokoto metropolis) and 10 trained research assistants collected the data over the first three weeks.

*Qualitative data*: the principal researcher moderated the FGD and was assisted by two trained research assistants who helped keep the time and take notes respectively.

### Data management

*Quantitative data*: the questionnaires were manually checked for completeness daily as research assistants returned them from the field. Data was entered into and analyzed using Epi info 7. Mean and standard deviation of continuous variables was obtained. Tabulation of frequencies and percentages of categorical variables was also done. To assess the perception of respondents, each correct response was allocated one (1) mark while a wrong, don't know or no response was scored zero. The perception score was calculated by dividing the total score obtained by the respondents by the total score obtainable and multiplying the answer by 100. Bivariate analysis (chi square test) was used to test the significance of associations between categorical variables and T test was used between continuous variables.

*Qualitative data*: narrative synthesis was used for findings gotten from the qualitative component of the study (FGD).

**Ethical consideration:** Ethics approval was obtained from the ethics and research committee of Kebbi state Ministry of Health in Birnin Kebbi, Nigeria. Informed consent was gotten from each respondent before they were enrolled into the study. The respondents were informed about the objectives of the research, potential risks and benefits associated with involvement in the research. They were assured of confidentiality of study findings and were freely allowed to decide whether to participate or opt out of the study voluntarily at any stage.

**Limitation of the study:** There is a possibility of deliberate misinformation by the respondents. This was minimized by educating the respondents on the importance of the research work and assuring them of information confidentiality. They were allowed to respond to the questionnaires individually in a semi-private area to encourage truthful responses.

## Results

**Results of the quantitative study:** Three hundred and forty two (342) correctly filled questionnaires were retrieved for analysis giving a response rate of 96.9%. Majority (71.1%) of the respondents' were aged 15-19 years with mean age 15.9 ± 2.4 years. Most of the respondents' were Males (88.6%), Hausa/Fulani (85.4%), Muslims (94.7%) and single (98.8%) (Table 1). About one third (32.2%) of respondents reported having ever smoked cigarettes while 20.8% currently smoke cigarettes. Seventeen percent (17.3%) of those who reported having ever smoked started smoking before the age of 10 years. Almost a quarter (23.6%) of smokers smoked cigarettes for more than 5 days in the last one month and 6.4% of them use more than 5 sticks of cigarettes daily. About thirty one percent (30.9%) of respondents bought their cigarettes themselves while 18.2% of them got theirs from friends. Almost half of smokers (49.6%) said they have never been refused cigarettes by vendors on account of their age. Almost twenty percent (19.5%) of respondents either smoke or feel like smoking cigarettes first thing in the morning (Table 2). The prevalence of cigarette smoking was statistically significantly higher among the 15-19 year olds when compared to the 10-14 year olds and also among the males when compared to females (Table 1). Majority of all the respondents (84.1%) had the perception that cigarette smoking was harmful and 70.8% also perceived that other smokers' cigarette smoke was dangerous to their health. There was no statistically significant difference between the proportion of smokers and non-smokers with this perception. Females who smoke cigarettes were perceived by fellow smokers to have more friends than females who do not smoke and this finding was statistically significantly different from how non-smokers viewed number of friends that female smokers have (p<0.008). Both male and female smokers were perceived by smokers to be more attractive than non-smokers. This perception was statistically significantly different from the perception non-smokers had about smokers (p<0.005 & p<0.001 respectively) (Table 3). The mean perception score of smokers was statistically significantly lower than that of non-smokers (p<0.005) (Table 3).

**Table 1:** Socio-demographic characteristics of respondents stratified by smoking status (n=342)

Variable	Total (%)	Ever smoked cigarette n=342		Test statistics
		Yes (%)	No (%)	
**Age group (years)**				χ^2^ = 16.30, p < 0.005
10-14	99 (28.9)	16 (16.2)	83 (83.8)	
15-19	243 (71.1)	94 (38.7)	149 (61.3)	
**Sex**				χ^2^ = 9.65, p < 0.005
Male	303 (88.6)	106 (35)	197 (65)	
Female	39 (11.4)	4 (10.3)	35 (89.7)	
**Tribe**				χ^2^ = 5.41, p < 0.144
Hausa /Fulani	292 (85.4)	97 (33.2)	195 (66.8)	
Yoruba	16 (4.7)	1 (6.3)	15 (93.7)	
Igbo	7 (2.0)	2 (28.6)	5 (71.4)	
Others	27 (7.9)	10 (37)	17 (73)	
**Religion**				χ^2^ = 0.167, p < 0.683
Islam	324 (94.7)	105 (32.4)	219 (67.6)	
Christian	18 (5.3)	5 (27.8)	13 (72.2)	
**Occupation**				χ^2^ = 1.77, p < 0.622
Artisan	121 (35.4)	38 (31.4)	23 (68.6)	
Any menial job	63 (18.4)	24 (38.1)	39 (61.9)	
Hawking	69 (20.2)	23 (33.3)	46 (66.7)	
Unemployed	89 (26.0)	25 (28.1)	64 (71.9)	
**Marital status**				χ^2^ = 0.09, p < 0.758
Single	338 (98.8)	109 (32.3)	229 (67.7)	
Married	4 (1.2)	1 (25.0)	3 (75.0)	

**Table 2:** Prevalence of cigarette smoking among respondents (n=342)

Variable	Frequency n=342	Percent (%)
**Ever smoked cigarettes (n = 342)**		
Yes	110	32.2
No	232	67.8
**Currently smoke cigarettes (n = 342)**		
Yes	71	20.8
No	271	71.2
**Age at first cigarette smoke (years) (n = 110)**		
≤ 10	19	17.3
11-13	32	29.1
14-16	45	40.9
≥ 17	14	12.7
**Number of days smoked in last month (n = 71)**		
1-5 days	45	63.4
> 5 days	26	36.6
**Number of cigarettes usually smoked daily (n = 71)**		
< 1 cigarette stick	15	21.1
1 daily	22	31.0
2-5 cigarettes daily	26	36.6
> 5 cigarettes daily	8	11.3
**Method of obtaining cigarettes (multiple responses allowed) (n = 71)**		
Bought them in a store	34	47.9
Got them from friends	20	28.6
An older person gave them to me	11	15.5
Gave someone money to buy it for me	10	14.1
Stole them	1	1.4
**Experience refusal to be sold cigarettes due to young age (n = 71)**		
Yes	14	19.7
No	57	80.3
**Ever smoke cigarettes or feel like smoking first thing in the morning (n = 71)**		
Yes	14	19.7
No	57	80.3
**Usage of other Tobacco products (n = 71)**		
Yes	23	32.4
No	48	67.6

**Table 3:** Respondents’ perception about cigarette smoking and their smoking status (n=342)

Variable	Total (%) n=342	Ever smoked		Test statistics
		Yes	No	
**Cigarette smoking is harmful to health**				χ^2^ = 5.240, p < 0.073
Yes	285 (84.1)	93 (32.6)	192 (67.4)	
No	37 (10.9)	8 (21.6)	29 (78.4)	
Not sure	17 (5.0)	9 (52.9)	8 (47.1)	
**Other smokers cigarette smoke is harmful**				χ^2^ = 3.616, p < 0.164
Yes	242 (70.8)	74 (30.6)	168 (69.4)	
No	63 (18.4)	19 (30.2)	44 (69.8)	
Don’t know	37 (10.8)	17 (45.9)	20 (54.1)	
**Difficulty associated with quitting smoking**				χ^2^ = 3.688, p < 0.158
Yes	247 (72.2)	84 (76.4)	163 (70.3)	
No	65 (19.0)	21 (19.1)	44 (19.0)	
Not sure	30 (8.8)	5 (4.6)	25 (10.8)	
**Boys who smoke have more or less friends than non-smokers**				χ^2^ = 2.145, p < 0.342
More friends	146 (42.7)	51 (46.4)	95 (41.0)	
Less friends	159 (46.5)	45 (40.9)	114 (49.1)	
No difference	37 (10.8)	14 (12.7)	23 (9.9)	
**Girls who smoke have more or less friends than non-smokers**				χ^2^ = 9.578, p < 0.008
More friends	97 (28.4)	36 (37.1)	61 (62.9)	
Less friends	222 (69.4)	61 (27.5)	161 (72.5)	
No difference	23 (6.7)	13 (56.5)	10 (43.5)	
**Comfort level of smokers at social gatherings**				χ^2^ = 2.455, p < 0.293
More comfortable	129 (37.7)	48 (43.6)	81 (34.9)	
Less comfortable	156 (45.6)	46 (41.8)	110 (47.4)	
No difference	57 (16.7)	16 (14.6)	41 (17.7)	
**Boys who smoke are more or less attractive**				χ^2^ = 24.277, p < 0.005
More attractive	46 (13.4)	28 (60.9)	18 (39.1)	
Less attractive	243 (71.1)	61 (25.1)	182 (74.9)	
No difference	53 (15.5)	21 (39.6)	32 (61.4)	
**Girls who smoke are more or less attractive**				χ^2^ = 19.773, p < 0.001
More attractive	27 (7.9)	17 (63.0)	10 (37.0)	
Less attractive	278 (81.3)	75 (27.0)	203 (73.0)	
No difference	37 (10.8)	18 (48.6)	19 (51.4)	
**Mean perception score**	76.40 ± 21.72	64.73 ± 24.60	81.94 ± 17.76	**p < 0.005, t = -6.57

**Results of the qualitative study:** Eight (8) FGDs were conducted i.e 5 among the non-smokers and 3 among the smokers. The findings that emerged from the focus group discussion were centered on: Respondents' perception about; cigarette smoking, smokers, harmful and beneficial effects of smoking.

**Respondents' perception about cigarette smoking:** There were two major/divergent views. The discussants in the FGDs done among cigarette smokers portrayed cigarette smoking in the good light. Majority of the respondents saw nothing wrong with cigarette smoking but rather as an attitude that every "REAL MAN" should imbibe. Their responses include: *"It is a necessary evil that symbolizes transition from childhood to manhood. One imbibes it so that he/she can face the challenges life brings headlong". "The young should try to avoid it". "Smoking is a status symbol, if you do not smoke then you are not yet a man". "Smoking makes you more attractive, gives you more friends and connections".* Most discussants in the non-smokers groups saw smoking as a bad habit that should be discouraged. Their responses include: "Smoking should be stopped, it causes harm". "I dislike cigarette smoking"*. "I do not like the smell that come out of cigarette smoke, it make me feel like vomiting".*

**Respondents' perception of cigarette smokers:** Most discussants in the smokers group perceived smokers as responsible, motivated and cool headed people who are willing to face challenges as they present themselves. The responses include: *"They impress me, they make friends". "Some people do not like them". "They are people who are always happy and willing to help". "They are cool-headed people who always have friends around them"* . Most discussants in the non-smokers group perceived smokers as being rascals, social deviants and frustrated individuals. The responses include: *"He is a fool, he is not wise and they usually want to impress someone". "They are rascals, cheat, thieves, and law breakers". "They are stubborn people, abusive, have bad friends". "They lack any form of menial job so they keep themselves busy with bad friends and so become smokers themselves". "They are losers, stupid and may God help them in stop if not may end up in jail".*

**Respondents' perception about harmful and beneficial effects of smoking:** Most of the respondents in the smokers group were of the opinion that smoking had more beneficial effects compared to harmful effects and that informed their choice to smoke. The responses include: *"Smoking makes you high, it relieves you of any stressful circumstance or problem you may be passing through and I cannot think of any dangerous effect associated with it". "If you do not smoke then how can you calm down with all this problems surrounding us? How do we feed? Where do we find a job to do?". "They say smoking is not good oh. But, whenever I smoke it helps me calm down, I have more friends now that I smoke and smoking makes me happier afterwards"* . Respondents in the non-smoking group were of the opinion that smoking had no beneficial effect but rather a lot of harmful effects. Responses include: *"Smoking has no benefits". "Smoking affects the lungs, heart, and chest pain. Those who smoke more are affected the most". "Smokers' liver becomes bad, they vomit blood and may die from it. The effects occur faster among frequent smokers". "Smoking at best gives only a temporal feeling of relieve because I have tried it before but on the long haul there is no benefit except to make you continue to look for more money to buy more cigarettes. It can make you become a thief and it is addictive. It was God that delivered me". "I am told one can develop chronic cough and other diseases so how can it be beneficial". "Smoking is dangerous and all smokers die earlier so therefore smoking is harmful"* .

## Discussion

Cigarette smoking is a serious public health problem among adolescents in this environment. In this study involving 342 out of school adolescents, the lifetime prevalence of cigarette smoking is 32.2%. This figure is comparable to that found in a similar study among out of school adolescents in North-eastern Nigeria where the prevalence was 33.9% [34] but much higher than that among secondary school adolescents in southwestern Nigeria where the prevalence was 20.5%. Most other similar studies among adolescents within Nigeria and elsewhere were carried out among secondary school students and the prevalence was much lower ranging between 3.4% and 27% [8, 12, 16, 19]. The current smoking prevalence of 20.8% was also much higher in this study than in findings in other studies [8, 11, 31, 33]. This may be an early pointer to the fact that out of school adolescents are less frequently researched but may have a much higher burden of cigarette smoking than may have been previously considered. Therefore the burden of cigarette smoking among adolescents in Nigeria may have hitherto been grossly underestimated and the real problem much bigger than had been previously thought of. The prevalence of cigarette smoking was found to increase with age among the respondents and the finding was statistically significant. This finding is comparable to that observed in similar studies in southwestern Nigeria [10, 35]. Therefore the prevalence of chronic respiratory diseases and cancers would be high in these group of individuals in middle age with very high costs of medical care and significant productivity workforce losses at a time they ought to form the backbone of the productive workforce of the country if current trend is not reversed. Some studies have shown smoking to be a predominantly male habit [10, 19, 20]. In this study males were shown to be up to 10 times more likely to smoke compared to females though most of the respondents were males (84%). However, this may not be unrelated to the fact that male adolescents are known to be more adventurous and so therefore more prone to try out new things. In addition, the FGDs revealed that out of school children are vulnerable to a lot of problems such as: exposure to exploitation by "bad people", ignorance about a lot of life issues and exposure to substance abuse including cigarette smoking, being cheated and stolen from and eventually becoming cheats and thieves themselves among others. This is made worse by the absence of adolescent friendly centers (AFCs) in Birnin kebbi where adolescents can go to when troubled, confused or in need of psychological help or counselling instead of falling in the hands of bad people. The discussants however acknowledged that these are more common among males who usually go out to fend for themselves as the females are usually indoors most times.

Amongst the smokers, it was observed that almost a quarter (23.6%) smoke cigarettes more than 5 days every month. The proportion that reported smoking for more than 5 days in a month in a similar study in Sokoto [16] was much higher which portrays the rate at which adolescents are beginning to imbibe cigarette smoking as a way of life. Almost one third of smokers in this study smoke at least 2 sticks of cigarettes daily. This is similar to findings in Sokoto [16] but much higher than that found in Ethiopia [33]. This shows the intensity with which the adolescents are embracing cigarette smoking. One of every five cigarette smokers in this study said they crave to smoke and most times smoke first thing in the morning when they wake up. This is a pointer to the addictive tendencies smokers tend to have which eventually makes it difficult to quit smoking despite becoming aware of the dangers associated with it. A third of the smokers also use other tobacco substances asides cigarettes. This is higher than the proportion using tobacco in a similar study in Oyo state, Nigeria [31] but lower than that in a similar study in Benin city Nigeria [35]. This finding may further buttress the growing addiction this group of adolescents are developing for tobacco and its products. It is worth noting that the increasingly stressful environmental conditions and the wrong information adolescents are vulnerable to receive from sources that lack credibility [31] may contribute to adolescents poor decision making. Majority of the respondents knew that cigarette smoking was dangerous to health and there was no statistically significant difference in knowledge about harmful effect of smoking between smokers and non-smokers. The focus group discussions revealed that most smokers became aware about the dangers associated with cigarette smoking after the habit had been imbibed and the addictive nature to cigarettes made it difficult to stop smoking afterwards. The perception that cigarette smoking makes smokers more attractive than non-smokers which was found to be statistically significantly higher among smokers is an important finding which needs to be taken very seriously in view of the comparable awareness level about the harmful effects of cigarette smoking between smokers and non-smokers. It means that these adolescents are aware of the dangers associated with cigarette smoking but because their perceived benefits outweigh the perceived risks they rather indulge in cigarette smoking than abstain from it.

Findings from the FGDs also showed that majority of the smokers actually felt that the beneficial effects of smoking such as; calming them when stressed up, being happier after smoking, forgetting about their problems etc outweighed the harmful effects and hence rather smoked cigarettes. Findings in the smokers group also showed that they believed smokers had more friends, were the real men who could face challenges whenever they came. This was however contrary to findings in the FGD among non-smokers where respondents felt smokers generally had bad friends hence they have other social vices such as being rascals, stubborn, deviants and law breakers. Smokers felt females who smoked had more friends and were more attractive. This is not encouraging among adolescents because this perception may on the long run tend to negatively affect the smoking perception and prevalence of smoking among their girlfriends or spouses if nothing is done to stop the ugly trend. Smokers also felt males who smoked cigarettes were more attractive than non-smokers. This is another negative perception which smokers talked glowing about during the FGDs and they argued their point on the basis that smokers were calmer, more relaxed and more sociable. They felt these were the indices to measure being attractive. Though the non-smokers had contrary opinions and felt smokers were losers, stupid, unemployed etc. this wrong impression about the perceived advantages of smoking will need to be corrected if we must succeed in halting and begin to reverse the increasing smoking initiation among adolescents. It was also observed that there was an inversely proportional relationship between respondents perception about cigarette smoking and the tendency to be a cigarette smoker i.e the better the perception of a respondent about cigarette smoking and the dangers associated with it, the less likely the respondent was a smoker and vice versa. The mean perception score of smokers was statistically significantly lower than that of non-smokers. Though this finding was not as distinct in similar studies where the perception between smokers and non-smokers was comparable [10, 34]. This portrays the importance perception plays as it relates to reasons why people tend to imbibe certain habits while others do not.

## Conclusion

This study showed that the prevalence of cigarette smoking is quite high among out of school adolescents. It also showed that most smokers had lower risk perception about cigarette smoking compared to non-smokers. They justified their smoking behavior on the perceived transient benefits associated with cigarette smoking.

### What is known about this topic

- The mean cigarette smoking prevalence of secondary school adolescents is 9% with some studies reporting a range of 3.7% - 17.1%;
- Previous studies have shown that most secondary school adolescents believe that cigarette smoking is harmful to their health.

### What this study adds

- Prevalence of cigarette smoking is very high among out of school adolescents, much higher than is being suggested by studies conducted among secondary school adolescents;
- Most out of school adolescents that smoke cigarettes became aware of harmful effects of smoking after smoking initiation;
- They also had the impression that beneficial effects of smoking outweigh the harmful effects.
